# Dimerization of VirD2 Binding Protein Is Essential for *Agrobacterium* Induced Tumor Formation in Plants

**DOI:** 10.1371/journal.ppat.1003948

**Published:** 2014-03-13

**Authors:** Abhilash Padavannil, Chacko Jobichen, Yang Qinghua, Jayaraman Seetharaman, Adrian Velazquez-Campoy, Liu Yang, Shen Q. Pan, J. Sivaraman

**Affiliations:** 1 Department of Biological Sciences, National University of Singapore, Singapore; 2 X4 Beamline, Brookhaven National Laboratory, Upton, New York, United States of America; 3 Institute of Biocomputation and Physics of Complex Systems (BIFI), Joint-Unit IQFR-CSIC-BIFI, and Department of Biochemistry and Molecular and Cell Biology, University of Zaragoza, Zaragoza, Spain, and Fundacion ARAID, Government of Aragon, Zaragoza, Spain; University of California Riverside, United States of America

## Abstract

The Type IV Secretion System (T4SS) is the only bacterial secretion system known to translocate both DNA and protein substrates. The VirB/D4 system from *Agrobacterium tumefaciens* is a typical T4SS. It facilitates the bacteria to translocate the VirD2-T-DNA complex to the host cell cytoplasm. In addition to protein-DNA complexes, the VirB/D4 system is also involved in the translocation of several effector proteins, including VirE2, VirE3 and VirF into the host cell cytoplasm. These effector proteins aid in the proper integration of the translocated DNA into the host genome. The VirD2-binding protein (VBP) is a key cytoplasmic protein that recruits the VirD2–T-DNA complex to the VirD4-coupling protein (VirD4 CP) of the VirB/D4 T4SS apparatus. Here, we report the crystal structure and associated functional studies of the C-terminal domain of VBP. This domain mainly consists of α-helices, and the two monomers of the asymmetric unit form a tight dimer. The structural analysis of this domain confirms the presence of a HEPN (higher eukaryotes and prokaryotes nucleotide-binding) fold. Biophysical studies show that VBP is a dimer in solution and that the HEPN domain is the dimerization domain. Based on structural and mutagenesis analyses, we show that substitution of key residues at the interface disrupts the dimerization of both the HEPN domain and full-length VBP. In addition, pull-down analyses show that only dimeric VBP can interact with VirD2 and VirD4 CP. Finally, we show that only *Agrobacterium* harboring dimeric full-length VBP can induce tumors in plants. This study sheds light on the structural basis of the substrate recruiting function of VBP in the T4SS pathway of *A. tumefaciens* and in other pathogenic bacteria employing similar systems.

## Author Summary

*Agrobacterium tumefaciens* causes crown gall disease (tumors) in agriculturally important plant species. It initiates infection through its Ti plasmid, which integrates a portion of its own DNA (T-DNA) into that of the host genome. The T-DNA is bound to VirD2 relaxase, and this complex is required for the efficient translocation and integration of the T-DNA into the plant genome for tumor formation. Two additional proteins, among others, are also required for *Agrobacterium* tumorigenesis: VirD4-coupling protein (CP) and VirD2-binding protein (VBP). VBP is responsible for recruiting VirD2–T-DNA to VirD4 CP to help localize T-DNA to the Type IV Secretion System apparatus for transfer. However, it is still unclear how VBP recruits the complex to VirD4 CP. Here, we report the crystal structure and associated functional studies of the C-terminal domain of VBP. We show that the C-terminal domain is the dimerization domain of VBP and only dimeric VBP is functional and essential for the induction of tumor in plants. This study enhances the understanding of the role of VBP in recruiting VirD2–T-DNA in *A. tumefaciens* prior to its transfer into the host plant. This mode of action can be extended to other pathogenic bacteria employing similar secretion systems.

## Introduction

The Type IV Secretion System (T4SS) has an unmatched versatility among the seven different secretion systems known in bacteria. T4SS can translocate not only proteins but also DNA to phylogenetically diverse taxa, including many bacterial species and various eukaryotic cells, as well as import and export DNA from the extracellular milieu. The T4SS shares a common ancestry with bacterial conjugation systems [1], with three types of T4SS described to date: (1) conjugation systems, defined as machines that translocate DNA substrates to recipient cells by a contact-dependent process; (2) effector translocation systems, functioning to deliver proteins or other effector molecules to eukaryotic target cells; and (3) DNA release or uptake systems, which translocate DNA to or from the extracellular milieu [2].

The VirB/D4 secretion system of *Agrobacterium tumefaciens* is a typical example of a T4SS, which serves as both a conjugation and an effector translocation system. Conjugation systems within the T4SS facilitate the translocation of protein-DNA complexes from the bacterium into the cytoplasm of the host cell. For instance, *A. tumefaciens* translocates to the host cell a segment of the Ti (tumor inducing) plasmid between the right and left borders (T-DNA) in complex with a cytoplasmic relaxase protein (VirD2) [3]. Proteins involved in T-DNA processing and translocation are classified into three functionally distinct classes [4]. Class I constitutes proteins involved in the processing of the DNA intermediates, such as VirC1, VirC2, VirD1 and VirD2 relaxase [5]. VirC1 and VirC2 proteins assemble the relaxosomal complex at the border sequences of the Ti plasmid and initiate T-DNA processing [6], whereas VirD2 relaxase, when present in the relaxosomal complex, cleaves the bottom strand of the T-DNA, where it remains covalently attached to the 5′ end of the single-stranded T-DNA [7]. Class II comprises 11 VirB proteins that form the T4SS apparatus which is responsible for the translocation of the VirD2–T-DNA complex and the effector proteins into the host cell cytoplasm [4], [8]. Class III constitutes the coupling proteins (CP), which mediate the interaction between the substrate (VirD2–T-DNA complex) and the transport apparatus [9]. VirD4 CP, the coupling protein in the *A. tumefaciens* VirB/D4 system, is an inner membrane protein with a large cytoplasmic domain essential for the transfer of both the T-DNA strand and VirE2 to host cells [10]–[12]. However, the VirD2–T-DNA complex and VirE2 are translocated separately [7], [13], [14]. The translocated effector proteins— VirE2, VirE3 and VirF which are involved in nuclear targeting, import and integration of the T-DNA into the host genome—mediate the interactions between the VirD2–T-DNA complex and host cellular factors [1]. In particular, VirE2 coats single-stranded T-DNA and protects it from host cell nucleases [15], whereas both VirE2 and VirD2 carry the nuclear localization signals that aid in the nuclear import of the T-DNA. VirE2 also interacts with host cell proteins, such as VIP1 and VIP2 [16]. VIP1 and VIP2 localize to plant nuclei and probably facilitate delivery of the T-DNA complex to its site of integration [16] VirF, on the other hand, localizes in the host nucleus and targets VIP1 and VirE2 for proteolysis and thus uncoats the T-DNA from its cognate proteins. This uncoating mechanism is a crucial step that must occur prior to integration of the T-DNA in the host genome [17].

The VirD2–T-DNA complex forms in the bacterial cytoplasm, but there is no evidence to suggest that VirD4 CP can recruit the bulky substrate complex to the T4SS apparatus [18]. In 2007, Guo *et al.* reported the existence of a subset of proteins defined as ‘recruiting proteins’ that are involved in bringing the nucleoprotein substrate complex formed in the bacterial cytoplasm to the VirD4 CP. The VirD2-binding protein (VBP) was subsequently identified as a key protein belonging to this subset and was shown to recruit the VirD2–T-DNA complex to the VirD4 CP [8]. Site-directed mutagenesis experiments have indeed shown that the interaction between VBP and VirD2 is important for T-DNA transfer [8], with VBP interacting with both the VirD2–T-DNA complex and with several of the T4SS components independently, including VirD4 CP, VirB4 and VirB11. However, the molecular mechanism(s) by which VBP recruits the complex to VirD4 CP remains unknown.

*A. tumefaciens* is a gram-negative bacteria that causes crown gall disease (tumor formation) in over 140 species of dicots [19].Despite this negative role, researchers have started to exploit the ability of *Agrobacterium* to infiltrate and infect other organisms to create transgenic plants. Thus, given the importance of this bacterium in both crop infection and as a research tool, we sought to better understand how VBP recruits the protein-DNA complex to VirD4 CP by exploring the structure of VBP.

Here we report the crystal structure of the C-terminal domain of VBP along with its functional studies. We show that the two monomers of the asymmetric unit form a tight anti-parallel dimer, with the dimerization confirmed by solution studies. Moreover, we demonstrate that this C-terminal domain is the dimerization domain of full-length VBP. Pull-down analyses and plant virulence assays showed that only *Agrobacterium* harboring dimeric VBP can interact with the key proteins to induce tumor formation in plants. These studies broaden our understanding about the role of VBP in the VirD2–T-DNA transfer from *A. tumefaciens* to the plant cell.

## Results

### Overall structure

**Figure 1:**
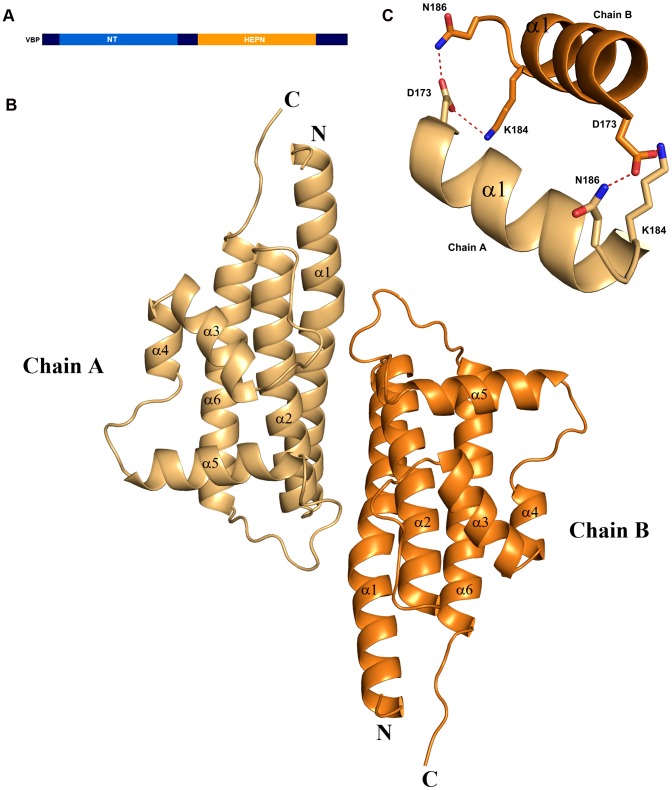
Overall structure. (A) Schematic representation of VirD2-binding protein (VBP) and its domains. Based on the sequence analysis, the N-terminal (aa 10–133) is predicted to be a nucleotidyltransferase (NT) domain and the C-terminal (aa 159–279) is predicted to be higher eukaryotes and prokaryotes nucleotide (HEPN) binding domain. (B) Structure of the HEPN domain of VBP. The asymmetric unit contains two monomers that form a tight dimer. Chain A is shown in light orange and Chain B is shown in deep orange. (C) Dimer interface of HEPN dimer. The contacts at the core of the HEPN dimer are shown.

We initially attempted to crystallize full-length VBP with intact N- and C-terminal domains (Figure 1A). Peptide-mass finger printing analysis on the crystals showed that only the C-terminal domain of VBP had crystallized. The boundaries of the crystallized protein were determined using N-terminal sequencing and mass spectrometric analysis. Subsequently, we generated a new construct that consisted of the C-terminal domain, and obtained crystals that diffracted up to 2.7 Å resolution. The structure was determined using the single wavelength anomalous diffraction (SAD) method (Table 1).The asymmetric unit consists of two monomers forming a tight dimer. Each monomer contains a six-helix bundle with three long anti-parallel α helices (α1, α2, and α6) that forms the major part of this domain, and three shorter helices (α3, α4 and α5) that stack at an angle to the long helices (Figure 1B, Figure S1A, S1B). The monomers are arranged as anti-parallel dimers with a large buried surface area of 1203 Å^2^ (or 14% of the total surface area of each monomer). The dimer is held together by tight interactions between α1 and α2 helices from both monomers (Figure 1C).

**Table 1:** Crystallographic statistics and refinement details.

	*SelMet SAD* #
**Data collection**	
Space group	P2_1_2_1_2_1_
Cell dimensions	
*a*, *b*, *c* (Å)	a = 63.10 b = 71.57 c = 83.42
α, β, γ (°)	90
Wavelength	0.97945
Resolution (Å)*	50.0-2.7(2.75-2.7)
*R*_sym_ a or *R*_merge_	0.05 (0.15)
*I*/σ*I*	22.4 (5.5)
Completeness (%)	99.7(99.3)
Redundancy	7.5(7.4)
**Refinement**	
Resolution (Å)	36.04-2.7
No. reflections	9671
*R*_work_ b/*R*_free_ c	0.218/0.28
No. atoms	
Protein	2428
Water	13
*B*-factors	
Protein	63
Water	57
R.m.s deviations	
Bond lengths (Å)	0.011
Bond angles (°)	1.372

# SAD – Single-wavelength anomalous diffraction.

a R_sym_ = Σ |I_i_−<I>|/Σ|I_i_| where I_i_ is the intensity of the i^th^ measurement, and <I> is the mean intensity for that reflection.

b R_work_ = Σ |F_obs_−F_calc_|/Σ|F_obs_| where F_calc_ and F_obs_ are the calculated and observed structure factor amplitudes, respectively.

c R_free_ = as for R_work_, but for 10.0% of the total reflections chosen at random and omitted from refinement.

Individual B-factor refinement was carried out.

*Values in parentheses are for highest resolution bin.

### Sequence and structural homology of VBP

PSI–BLAST searches of the non-redundant protein database using the full length VBP (gi|159141484) resulted in several hits of nucleotidyltransferase proteins from *Rhizobium*, *Sinorhizobium* and other species of *Agrobacterium*. Sequence-based predictions revealed that VBP has two domains: an NT_KNTase -like domain at the N-terminus and a HEPN (higher eukaryotes and prokaryotes nucleotide-binding) domain at the C-terminus. The NT (nucleotidyltransferase) domain is implicated in nucleotidyl transfer function, whereas the HEPN domain is implicated in nucleotide binding[20]. A DALI [21] search for the structural homologs of VBP C-terminal domain identified several proteins with a HEPN domain (Table S1); this confirmed that the C-terminal domain of VBP adopts a HEPN fold (hereafter referred to as HEPN domain). Notably, the HEPN domain of VBP aligns with the C-terminal domain of kanamycin nucleotidyltransferase from *Staphylococcus aureus* (PDB code: 1KNY) with an rmsd of 2.9 Å for the 104 Cα atoms. Although VBP has very low sequence identity (14 to 16%) with its structural homologs, it might have similar nucleotide binding and transfer function.

### VBP is a dimer

**Figure 2:**
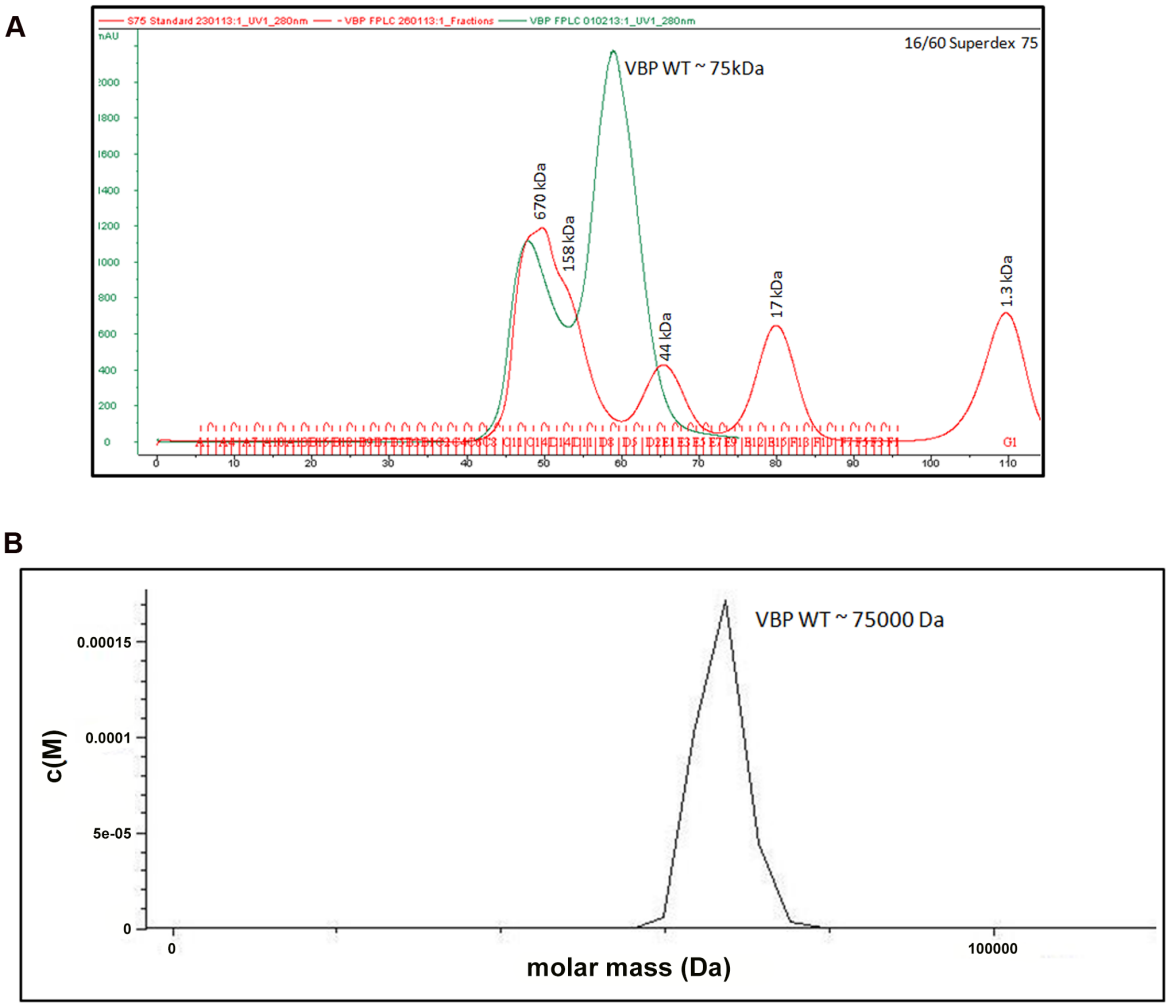
VBP is a dimer. (A) Gel filtration profile of VBP. Full-length VBP elutes as a single peak (in green) at an elution volume corresponding to an apparent molecular mass of 75 kDa. The molecular weight standard is shown in red. The peak at 670 kDa corresponds to aggregated VBP that elutes in the void. (B) Analytical ultra-centrifugation profile of VBP. The full-length VBP sediments as a single species at an apparent molecular mass of 75 kDa.

The crystal structure of the HEPN domain shows the presence of a tight dimer in the asymmetric unit. The molecular mass based on the sequence of VBP is 37.5 kDa (including the 6His tag). However, size-exclusion chromatography showed that VBP elutes as a single peak at an elution volume corresponding to an apparent molecular mass of 75 kDa (Figure 2A). Further, the analytical ultracentrifugation (AUC) analysis showed that VBP sediments as a single species corresponding to an apparent molecular mass of dimeric VBP (75 kDa) (Figure 2B). Taken together, these results show that VBP forms a homodimer in solution and likely functions as a dimer in the cell.

### HEPN domain of VBP is the dimerization domain

**Figure 3:**
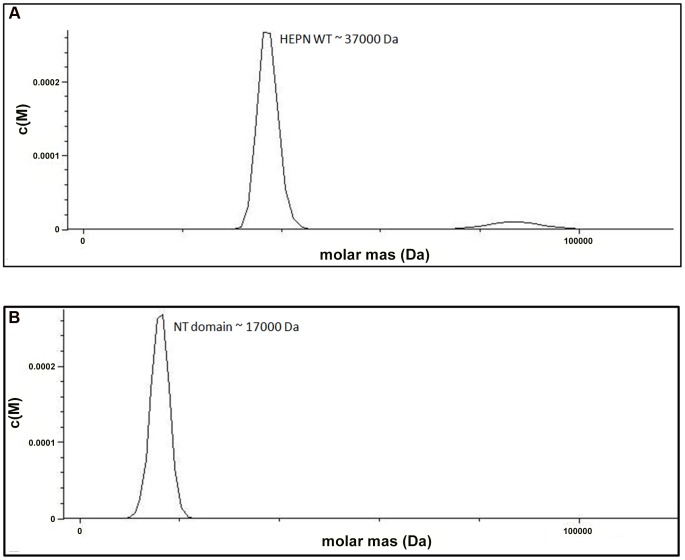
The HEPN domain of VBP is the dimerization domain. (A) Analytical ultra-centrifugation profile of the HEPN domain of VBP. The HEPN domain sediments as a major species at an apparent molecular mass of 37 kDa. (B) Analytical ultra-centrifugation profile of the NT domain of VBP. The NT domain sediments as a major species at an apparent molecular mass of 17 kDa. Abbreviations: HEPN, higher eukaryotes and prokaryotes nucleotide binding domain; NT, Nucleotidyltransferase domain.

We examined the role of the HEPN domain in VBP oligomerization by generating individual constructs for the N-terminal (NT) and C-terminal (HEPN) domains of VBP. The size-exclusion chromatography of the purified proteins showed that the NT domain elutes as a single peak at an elution volume corresponding to an apparent molecular mass of 16.8 kDa (NT as monomer), whereas the HEPN domain elutes as a single peak at an elution volume corresponding to an apparent molecular mass of 37 kDa (HEPN as a dimer) (Figure S2). These results were further confirmed using analytical ultracentrifugation experiments (Figure 3A, 3B), and are consistent with the crystal structure findings that show the presence of tight dimeric HEPN domains in the asymmetric unit. Taken together, these results suggest that HEPN domain is responsible for the dimerization of the full-length VBP.

### Substitution of key residues disrupts the dimerization

**Figure 4:**
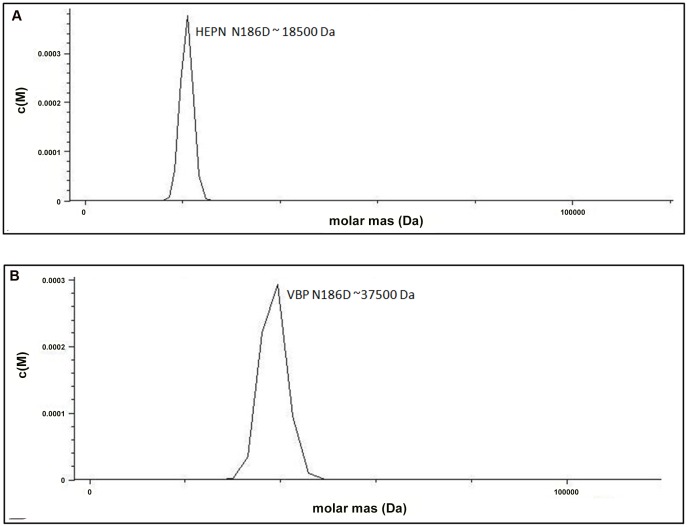
Substitution of key residues disrupts dimerization. (A) Analytical ultra-centrifugation profile of HEPN Asn186Asp domain of VBP. HEPN domain with Asn186Asp substitution sediments as a single species at an apparent molecular mass of 18.5 kDa. (B) Analytical ultra-centrifugation profile of VBP Asn186Asp. VBP with Asn186Asp substitution sediments as a single species at an apparent molecular mass of 37.5 kDa. Abbreviations: HEPN, higher eukaryotes and prokaryotes nucleotide (domain).

The structural analysis indicated that the HEPN dimer is held together by contacts throughout helices α1 and α2; in particular, residues Asp173, Lys184 and Asn186 of the HEPN domain play important roles in maintaining the dimer. Asn186 is located at the edge of a loop connecting the α1 and α2 helices of the dimer interface (Figure 1C). This residue, along with Lys184 and Asp173 of one monomer, is involved in hydrogen bonding contacts with Asp173 and Asn186, Lys184 of the second monomer. We found that a single substitution of Asp173Asn, Lys184Asp or Asn186Asp in the HEPN domain is sufficient to disrupt dimer formation, as verified by size-exclusion chromatography and analytical ultracentrifugation experiments (Figure 4A, Figure S3A, S3B and Figure S4).

Next, we verified the role of these key residues using full-length VBP. Single point substitutions of Asp173Asn, Lys184Asp or Asn186Asp in full-length VBP caused the dimer to break and the protein to elute as a single peak at an elution volume corresponding to an apparent molecular mass of 37.5 kDa (monomeric VBP). Further AUC analyses of full-length VBP bearing one of these point substitutions showed that the protein sediments as a single species at an apparent molecular mass corresponding to monomeric VBP (37.5 kDa) (Figure 4B, Figure S5A, S5B and Figure S6). The circular dichroism (CD) spectrum of the wild-type VBP, mutated VBP and HEPN domain suggested that these mutants have the same structural fold as the wild-type proteins (Figures S7A, S7B). These results indicate that the hydrogen bonds at the dimeric interface play a key role in maintaining an intact dimeric HEPN domain and reiterate the involvement of the HEPN domain in VBP dimerization.

### VBP binds to VirD2 and VirD4 CP as a dimer

**Figure 5:**
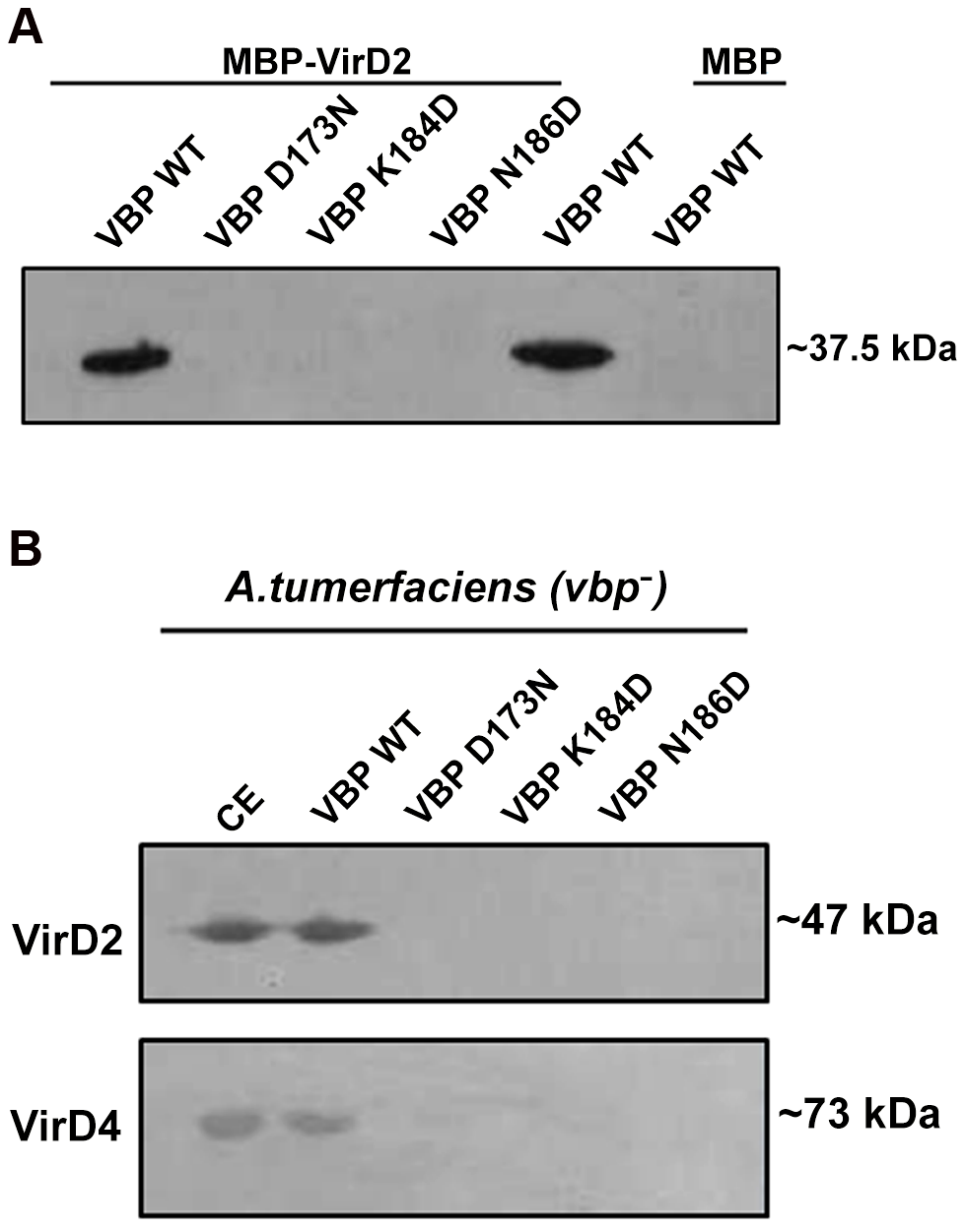
Pull down assays. (A) MBP/MBP-VirD2 bound to amylose resin was incubated overnight with 6His-VBP, followed by washes. The final beads were resolved on a 12.5% SDS gel, transferred to a PVDF membrane and treated with anti-His monoclonal antibody (1∶10,000). 6His-VBP was loaded into the lane 5 as a reference. The VBP species used include: lane 1. Wild-type (WT) VBP; lane 2.VBP D173N; lane 3.VBP K184D; lane 4. VBP N186D; lane 5. VBP wild-type loaded on to the gel for reference; .lane 6. VBP passed through MBP bound to amylose beads. (B) 6His-VBP/substituted 6His-VBP bound to Ni-NTA metal affinity resin was incubated with freshly prepared *A. tumefaciens* crude extracts. After incubation at 4°C for 1 h, the resin was washed four times. The bound complex was eluted with 250 mM imidazole. The eluted protein was resolved on SDS-Gel, transferred to a PVDF membrane and the protein was detected using protein (VirD2 and VirD4 CP) specific monoclonal antibodies. The VBP species used include: lane 1. Crude extract loaded for reference; lane 2. WT VBP; lane 3.VBP D173N; lane 4.VBP K184D; lane 5. VBP N186D.

VBP is the key recruiting protein that binds to the VirD2–T-DNA complex and brings it into contact with VirD4 CP for subsequent translocation to the host cell [8]. Thus, we sought to verify the binding property of VBP with VirD2 and VirD4 CP using pull-down assays. Using an *in vitro* pull-down assay with recombinant proteins (6His-VBP and MBP-VirD2), we showed that wild-type VBP binds to VirD2, whereas VBP with one of these aforementioned single point substitutions—Asp173Asn, Lys184Asp or Asn186Asp (which results in monomeric VBP)—does not bind VirD2 (Figure 5A). To confirm these results, we used His-VBP and substituted His-VBP (with (Asp173Asn/Lys184Asp/Asn186Asp mutations) as bait and pulled down the VBP-interacting proteins from a *vir* gene-induced *Agrobacterium* null mutant that lacks VBP: GMV123 (A triple *vbp* null mutant strain GMI9017*Δvbp2Δvbp3* (for which all three existing *vbp* genes were knocked out)). WT His-VBP was able to pull down VirD2 and VirD4 CP, whereas substituted VBP could not pull down either of these proteins. These results indicate that the dimeric nature of VBP is important for its interaction with VirD2 and VirD4 CP (Figure 5B).

### VBP functions as a dimer *in vivo*

**Figure 6:**
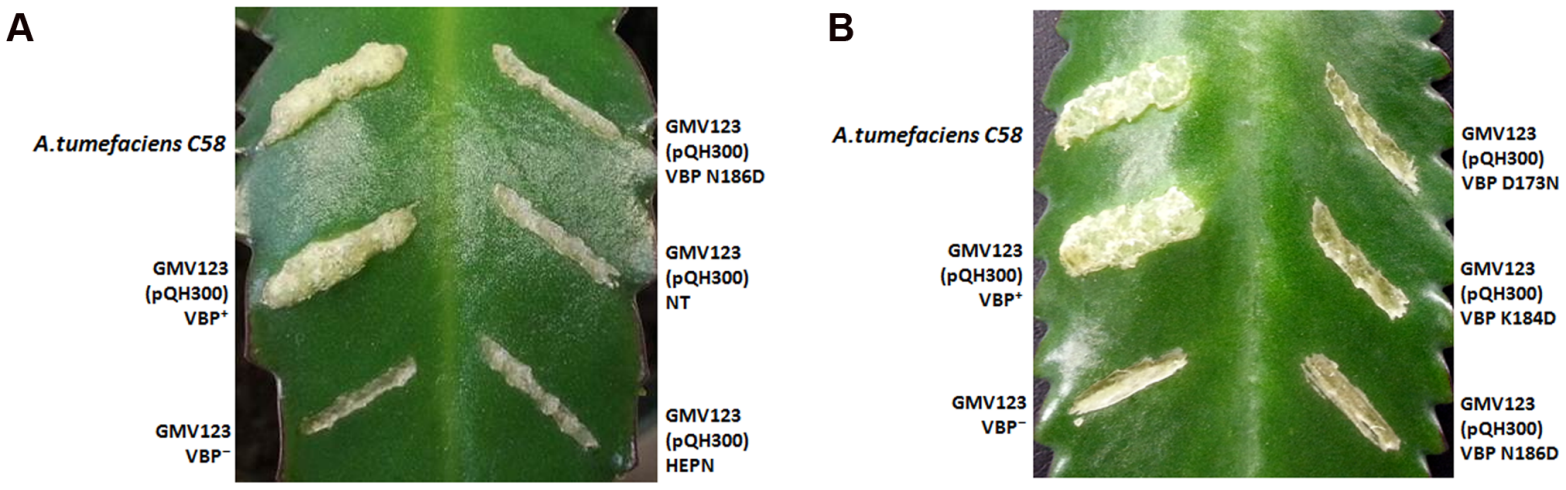
VBP is a functional dimer. The effect of VBP mutations on tumorigenesis. *A. tumefaciens* strains were grown in MG/L medium at 28°C overnight. The cell density was adjusted to 10^8^cells/ml. This cell suspension (5 µl) was inoculated into each wound site on the leaves of *Kalanchoe* plants. The tumors were photographed at 25 days (A) The wounds on the *Kalanchoe* leaf were inoculated with *A.tumefaciens* WT strain or GMV123 strain complemented with plasmid expressing VBP WT, VBP N186D, NT domain or HEPN domain. Only wounds inoculated with *A.tumefaciens* WT strain or GMV123 strain complemented with plasmid expressing VBP WT showed tumor, other wounds showed no tumor clearly indicating that only *Agrobacterium* harboring full length VBP can induce tumor. (B) The wounds on the *Kalanchoe* leaf were inoculated with *A.tumefaciens* WT strain or GMV123 strain complemented with plasmid expressing VBP WT, VBP N186D, VBP D173N or K184D. Only wounds inoculated with *A.tumefaciens* WT strain or GMV123 strain complemented with plasmid expressing VBP WT showed tumor, other wounds showed no tumor clearly indicating that only Agrobacterium harboring full length dimeric VBP can induce tumor. The particular mutants are labeled at the respective scars.

The observed dimeric nature of VBP in solution and in the crystal structure prompted us to verify the functional state of VBP inside the cell using a plant virulence assay. GMV123 (*vbp* KO strain of *A. tumefaciens*) was complemented with pQH300 plasmid harboring substituted constructs for a functional *vbp* gene, a gene expressing the HEPN domain, or a gene expressing the NT domain. We found that null mutants transformed with a plasmid expressing VBP were able to cause a tumor-like phenotype in the wild-type plants (Figure 6A and 6B and Figure S8). Strains expressing a substituted VBP (Asp173Asn/Lys184Asp/Asn186Asp) or one of the other deletion mutants (pQH-NTD or pQH-HEPN) did not cause tumors (Figure 6A and 6B and Figure S8). These results indicate that VBP functions as a dimer in the cells and that full-length VBP is required for tumor formation in plants.

### Interaction of VBP with ATP

**Figure 7:**
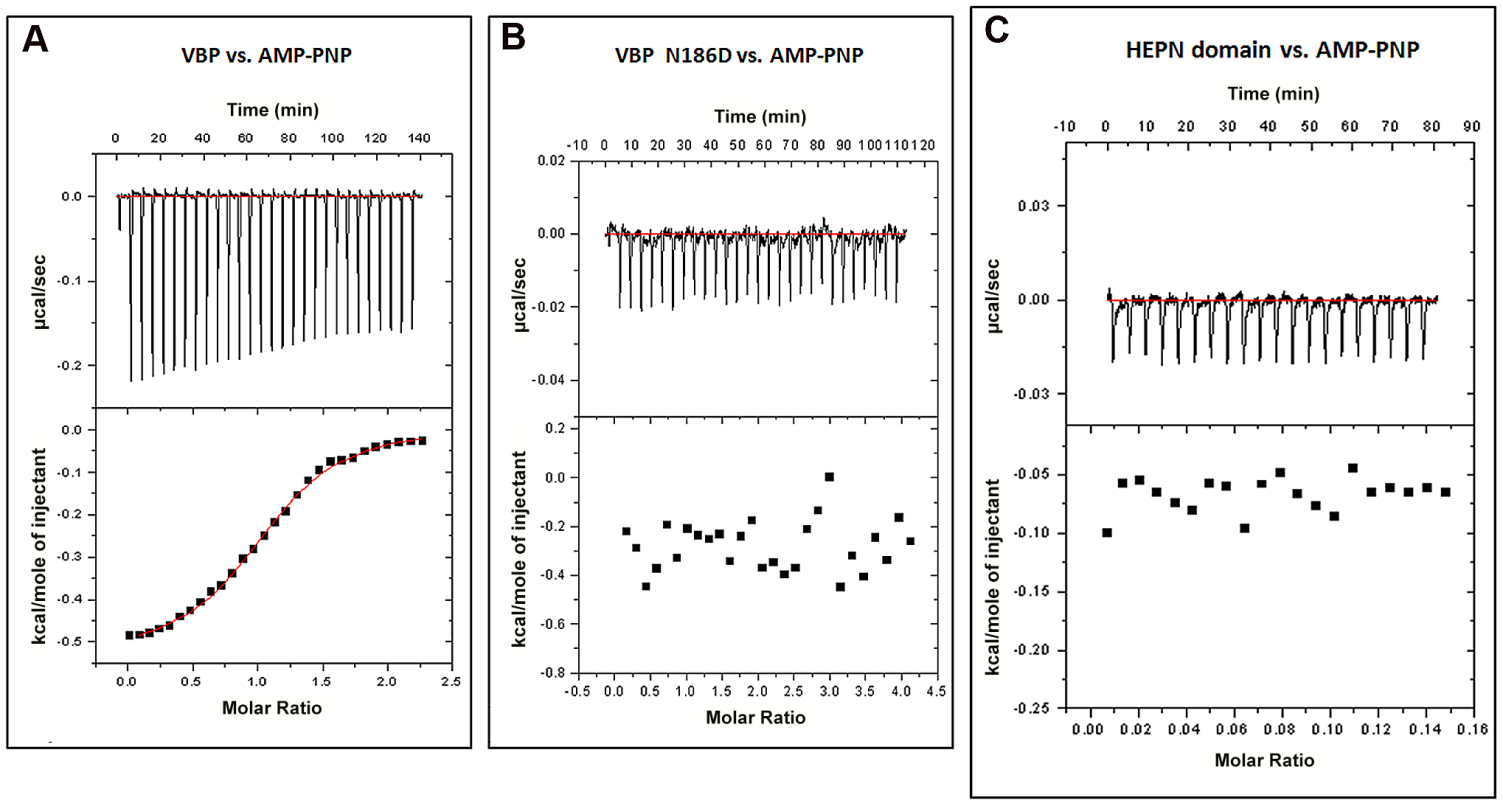
Interaction of VBP with AMPPNP (ATP analog) by isothermal titration calorimetry (ITC). Representative ITC assays are shown. The upper part of each panel shows the thermogram (thermal power *vs.* time) after baseline correction and the bottom part of each panel shows the binding isotherm (normalized heat *vs.* molar ratio of reactants). (A) Calorimetric titration for VBP. (B) Calorimetric titration for VBP N186D. (C) Calorimetric titration for HEPN.

Proteins homologous to VBP are predicted to bind ATP [22]. A previously reported structure of kanamycin nucleotidyltransferase (PDB code: 1KNY), a structural homolog of VBP, in complex with an ATP analog and kanamycin, shows that the nucleotide binding pocket involves residues from the N-terminal domain of one monomer and the C-terminal domain of the second monomer. We verified the ATP binding property of VBP using isothermal titration calorimetry (ITC) (Figure 7A and Table S2). The ITC analysis showed that VBP binds to the ATP analog (AMPPNP) (K_d_ = 2.0 µM), whereas VBP with the three point substitutions described above does not bind to AMPPNP (Figure 7B, Figure S9A and S9B), indicating that only dimeric VBP binds to ATP. Further, using ITC experiments, we sought to verify whether the HEPN domain alone can bind nucleotides (Figure 7C). Our results indicated that the HEPN domain alone cannot bind nucleotides. We infer that, similar to the kanamycin nucleotidyltransferase, the ATP binding in VBP might involve both N-terminal and C-terminal domains. Notably, the structure of the kanamycin nucleotidyltransferase (PDB code: 1KNY) complexed with a nucleotide analog and kanamycin shows that the two monomers of the dimer interact in an anti-parallel fashion to form the ATP binding pocket. Similarly, the structure of the HEPN domain from VBP shows that the two HEPN monomers form a tight dimer in which the monomers run in anti-parallel. Although the relative orientation of monomers in the dimers of both proteins is not the same, both proteins might have a similar ATP-binding mechanism.

## Discussion

*A. tumefaciens* affects more than 140 species of dicots [19], instigating infection through the efficient translocation of the VirD2–T-DNA complex [2], a prerequisite for the integration of T-DNA into the plant genome and eventual tumor formation in plants [23]. The T-DNA is a segment of the Ti plasmid that encodes most of the proteins that are involved in VirD2–T-DNA complex translocation and T-DNA integration into the host genome, with each protein catering to a particular stage of the translocation process. The VirD4 CP is known to couple the VirD2–T-DNA complex mediated by VBP as a recruiting complex (VBP: VirD2–T-DNA) to the T4SS secretion apparatus. VBP is thus a key component of the recruiting complex [8].

Based on the sequence analysis, it has been predicted that the NT domain of VBP belongs to the DNA polymerase β superfamily of proteins [21]. This superfamily includes nucleotidyltransferases that catalyze nucleotidylation of proteins in yet unidentified pathways [21]. Similarly the C-terminal of VBP is predicted to have the HEPN domain [24]. VBP interaction with VirD2, and several other energizing components of the T4SS (VirD4, VirB4 and VirB11) [13], makes it difficult to predict the exact nucleotidylation site of the protein.

**Figure 8:**
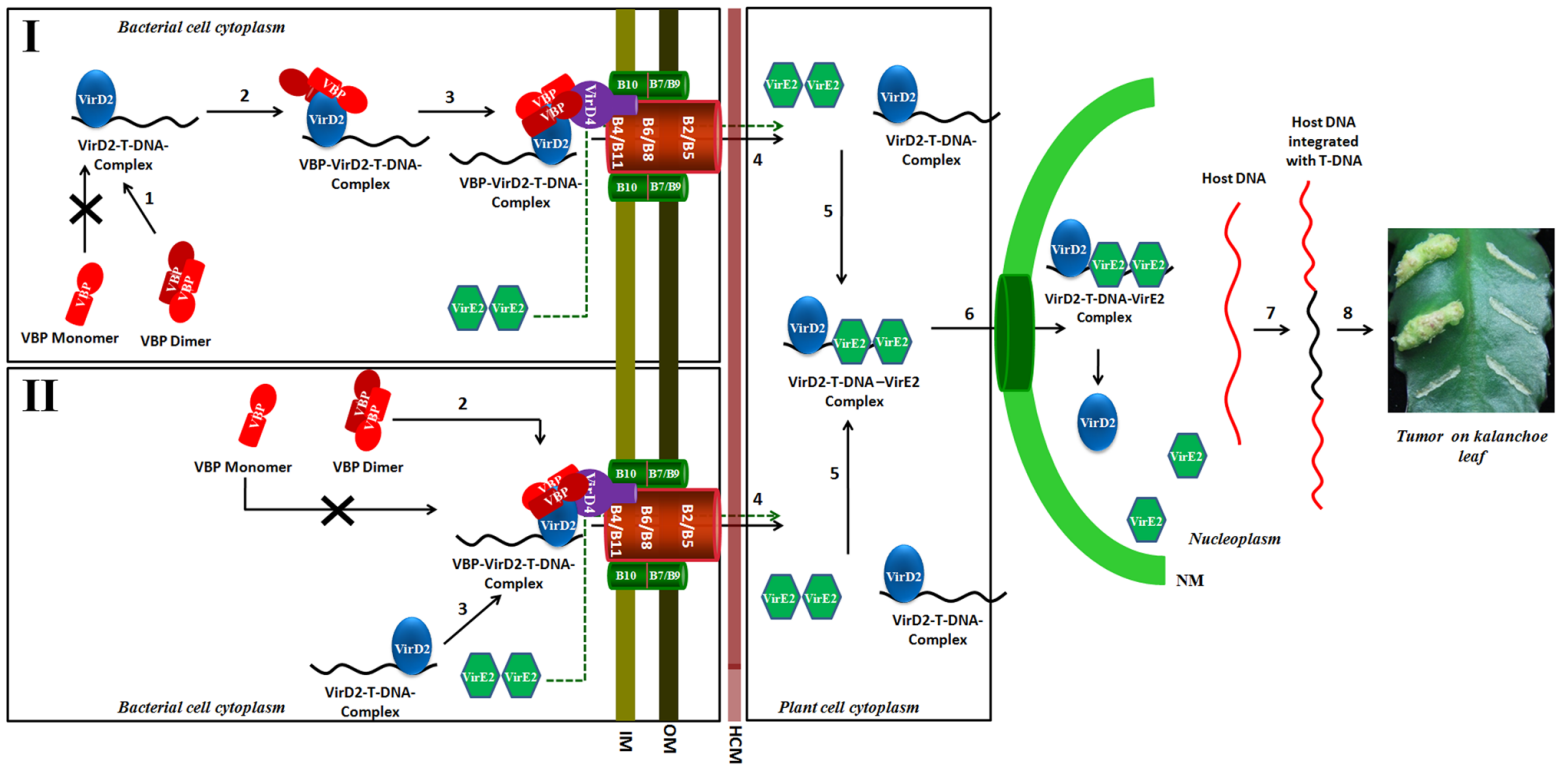
Schematic representation shows the induction of tumors in plants by *Agrobacterium tumefaciens* and the role of VBP. Our experiments show that only dimeric VBP can bind to VirD2 or VirD4 CP. We propose that the VirD2–T-DNA complex could possibly be recruited to the T4SS apparatus by two different mechanisms. Mechanism I: Dimeric VBP binds to VirD2 (steps 1 and 2), it recruits the VirD2–T-DNA complex to the (VirD4 CP) T4SS apparatus which constitutes the 11 VirB proteins (step 3). Mechanism II: Dimeric VBP binds to VirD4 CP (step 2). VBP acts as a docking station to recruit the VirD2–T-DNA complex (step 3). Once recruited to the T4SS apparatus, the VirD2–T-DNA complex is translocated into the host cell cytoplasm (step 4). It is yet unclear whether VBP is translocated or not [8]. VirE2 is one among the several effector proteins that are translocated through the T4SS. Inside the host cell, VirE2 coats the single-stranded T-DNA to form the VirD2–T-DNA-VirE2 complex (step 5). Certain host cytoplasmic proteins recognize and bind to the nuclear localization signals on VirD2 and VirE2 and translocate the VirD2–T-DNA:VirE2 complex to the nucleus (step 6) [35]. Inside the nucleus, VirD2 and VirE2, along with a plethora of host proteins, help the T-DNA to integrate with the host DNA (step 7) [36]. The integrated T-DNA modulates the host cell process to enable the bacterial colonization and growth, which leads to the formation of tumor (step 8). IM: Inner membrane; OM: Outer membrane; HCM: Host cell membrane; NM: Nuclear membrane.

In this study, we sought to analyze the structural and functional aspects of the C-terminal domain of VBP. We show that this domain adopts a HEPN fold and facilitates the dimerization of VBP, forming tight anti-parallel dimers. The structural similarity observed between the HEPN domain of VBP and kanamycin nucleotidyltransferase, as well as the results from our ITC experiments, suggest that a nucleotide binding pocket is formed by the dimeric interface in this protein. Furthermore, our pull-down assays show that only dimeric VBP can bind VirD2 and VirD4 CP and that dimerization of VBP is essential for *A. tumefaciens*-induced tumor formation in plants (Figures 5, 6 and 8).

Previously, we showed that VBP has independent interactions with VirD4 CP, VirB4 and VirB11 ATPases [8]. VirD4 CP has a prominent cytoplasmic domain, besides its membrane embedded domain [1], whereas VirB4 and VirB11 have both membrane and the cytoplasmic regions [25]. Our previous studies have shown that VBP is a cytoplasmic protein that localizes at the poles only in the presence of T4SS [8]. This is probably because of the interaction between VBP and the cytoplasmic regions of the T4SS proteins, particularly VirD4 CP, VirB4 and VirB11. Furthermore, the polar localization of VBP does not depend on the presence of VirD2 [8].

Although VirD2 is a cytoplasmic protein, it localizes to the poles in the presence of VBP and T4SS apparatus, but remains in the cytoplasm in the absence of VBP, irrespective of the presence of T4SS apparatus [8]. This indicates that when T4SS is present, VBP binds to the cytoplasmic region of VirD4 CP either independently or as a complex with VirD2, when it is available. In light of this finding, we propose that either VBP binds to the VirD2–T-DNA complex and recruits it to the VirD4 CP, or VBP initially binds to VirD4 CP proteins at the cytoplasmic region and serves as a docking station for the VirD2–T-DNA complex (Figure 8). Using a transfer DNA immunoprecipitation (TrIP) assay, Cascales *et al.* elegantly showed that T-DNA recruited to VirD4 CP is transferred to VirB6 through VirB11 [7] and that VirB4 and VirB11 ATPases interact to drive the export of T-DNA [7].

The present study sheds light on the role of VBP in the VirD2–T-DNA complex translocation in VirB/D4 T4SS of *A. tumefaciens* and other similar bacterial system that use the Type IV secretion conjugation systems.

## Methods

### Plasmid and strain construction

The strains and plasmids used are given in Table S3. Intact *vbp* and *vird2* genes were amplified from *A. tumefaciens* C58 plasmid and Ti plasmid, respectively. These genes were then cloned to pET32a (Novagen; Madison, WI, USA) and pRSET (Invitrogen, Carlsbad, CA, USA) vectors, respectively. N-terminal nucleotidyltransferase (NT) domain and C-terminal HEPN domain constructs were created using specific primers that amplify these regions and were cloned into pGEX-6p1 (GE Healthcare; Buckinghamshire, UK). Site-specific mutations in *vbp* were introduced by overlapping PCR, as described previously [26]. Each construct was verified by DNA sequencing. A fragment of *virF* cassette cloned from pTiBo542 (GenBank: DQ058764.1) was inserted into the SphI–ApaI site on pCB301 [27]. The *virF* coding sequence was substituted with a multiple cloning site, resulting in pQH300.

### Protein expression and purification

The plasmid pET32a-*vbp* was transformed into *E. coli* BL21 (DE3) cells and was grown in LB broth at 37°C overnight. The overnight culture was transferred into 1 L of LB broth and the protein expression was induced at an absorbance of 0.6 with 350 µM IPTG for 20 h at 20°C.Cells were harvested and lysed in lysis buffer (20 mM Tris-HCl, pH 8.0, 200 mM NaCl and 1 mM PMSF). Cell lysates were centrifuged and the supernatants transferred to affinity columns containing Ni-NTA agarose (Qiagen; Valencia, CA, USA), pre-equilibrated with the lysis buffer. The 6His-VBP bound to Ni-NTA was eluted with 400 mM imidazole following three wash steps to remove non-specific proteins. The eluted protein was purified through size-exclusion chromatography using a HiLoad 16/12075 Superdex75 gel filtration column (AKTA FPLC UPC-900 system, GE Healthcare) containing buffer (20 mMTris-HCl, pH 8.0, 200 mM NaCl, and 5% glycerol). The GST fusion proteins (GST-HEPN and GST-NTD) were expressed as described above using M9 media [28]. The fusion proteins were purified by affinity chromatography on GST-Sepharose resin, and the tags were removed by cleavage with PreScission proteases (GE Healthcare; Buckinghamshire, UK). The HEPN domain was additionally purified by size-exclusion chromatography in gel-filtration buffer (30 mM CHES pH 9.0, NaCl 200 mM, 5% glycerol). The NT domain was purified in the same way but using a buffer containing 30 mM Tris-HCl, pH 7.5 and 200 mM NaCl buffer.

### Crystallization and data collection

Initial crystallization conditions were identified by hanging drop vapor diffusion method using an index screen (Hampton Research, Aliso Viejo, CA, USA). Diffraction-quality crystals were obtained by equilibrating l.0 µl drop of protein (4 mg/ml) in 30 mM CHES, pH 9.0, 200 mM NaCl and 5% glycerol mixed with 1.0 µl of reservoir solution (8% (w/v) PEG 3350, 2% v/v tacsimate, 5% v/v 2-proponal, and 0.1 M imidazole) suspended over 1 ml of reservoir solution. Crystals grew in 1–3 days at 16°C. For data collection, 15% glycerol was added as a cryo-protectant and the crystals were flash-cooled in an N_2_ cold stream.

A complete single wavelength anomalous diffraction (SAD) [29] dataset was collected to 2.7 Å resolution at the synchrotron beamline X6A (National Synchrotron Light Source, Brookhaven National Laboratory, Upton, NY) using a Quantum4-CCD detector (Area Detector Systems Corp., Poway, CA). The datasets were processed and scaled using HKL2000 [30]. The crystals belonged to a P2_1_2_1_2_1_ space group. There were two monomers in the asymmetric unit corresponding to *Vm* = 2.49 Å^3^ Da^−1^ with a solvent content of 50.6%. The position of the selenium atoms were determined using the program Phenix-Autosol [31]. The obtained phases were further improved by density modification using RESOLVE [31]. Over 50% of the backbone atoms of the model were built by RESOLVE. The remaining residues were manually built using Coot [32] and subsequently refined using Refmac [33]. Refinement was continued until the R-value converged to 0.22 (R_free_ = 0.28) for reflections I>σ (I) to 2.7 Å resolution (Table 1). The model had good stereochemistry, with 99.3% residues within the allowed regions of the Ramachandran plot. Subsequently, the importance of the key residues at the dimeric interface was validated by structure-based *in vitro* studies, such as analytical ultracentrifugation and pull down assays, and *in vivo* plant virulence studies. Coordinates of HEPN domain of VBP have been deposited in the Protein Data Bank (http://www.pdb.org) under accession code 4NQF.

### Analytical ultracentrifugation

The oligomeric state of full-length VBP, HEPN and NTD domain of VBP and their mutants was investigated by monitoring the sedimentation properties of each protein in sedimentation velocity experiments. For these experiments, 400 µl of samples at 1 mg/ml in appropriate buffer were used, with the experiments carried out in duplicate. The sedimentation velocity profiles were collected by monitoring the absorbance at 280 nm. The samples were centrifuged at 40,000 rpm at 24°C in a Beckman Optima XL-I centrifuge fitted with a four-hole AN-60 rotor and double-sector aluminum centerpieces and equipped with absorbance optics. Eighty scans were collected and analyzed using the Sedfit program [34].

### Pull down assay

MBP-VirD2 bound to amylose resin (New England Biolabs, Ipswich, MA, USA) was incubated with purified 6His-VBP, with or without substitution at Asp173Asn/Lys184Asp/Asn186Asp. The beads were washed several times before resolving on 12.5% SDS-PAGE. For western blot analysis, the proteins were transferred to a PVDF membrane. 6His-VBP was detected by the addition of diluted anti-His antibody (Santa Cruz Biotechnology; Santa Cruz, CA, USA). The signal was detected using the Super Signal WestPico Chemiluminescent substrate (Pierce Biotechnology; Rockford, IL, USA) under the conditions recommended by the manufacturer. For pull down assay from *A. tumefaciens* crude extracts, *E. coli strain BL21* was used to produce 6His-VBP/substituted 6His-VBP as described above.6His-VBP/substituted 6His-VBP bound to Ni-NTA metal affinity resin was incubated with freshly prepared *A. tumefaciens* (*vbp* KO strain) crude extracts (2.5 mM MgCl2, 50 mM NaCl, 2 mM PMSF, 50 mM Tris-HCl, pH 7.4, 0.5% Triton X-100). After incubation at 4°C for 1 h, the resin was washed four times. The bound proteins were eluted with 250 mM imidazole. The eluted proteins were resolved on 12.5% SDS-PAGE. For western blot analysis, the proteins were transferred to a PVDF membrane. VirD2 and VirD4 CP in the eluent were detected by western blot using anti-VirD2 (1∶5000) and anti-VirD4 (1∶4000) antibodies. The signal was detected using the Super Signal WestPico Chemiluminescent substrate (Pierce Biotechnology; Rockford, IL, USA) under the conditions recommended by the manufacturer.

### Isothermal titration calorimetry

6His-VBP, with or without substitution of key residues, were purified in gel filtration buffer containing 30 mM Tris-HCl, pH 8.0 and 200 mM NaCl. ITC experiments were carried out using a VP-ITC calorimeter (MicroCal, LLC, Northampton, MA, USA) at 25°C using 0.01 mM VBP protein in the sample cell and 0.25 mM AMP-PNP in the injecting syringe. All samples were thoroughly degassed and then centrifuged to remove precipitates. With the exception of the first injection, 10 µl volumes per injection were used for different experiments. Consecutive injections were separated by 5 min to allow the peak to return to baseline levels. ITC data were analyzed with a single-site binding model using Origin 7.0 (Origin Lab Corp., Northampton, MA, USA) software.

### Circular dichroism spectrometry

Far UV spectra (260–190 nm) of VBP, HEPN, NTD domains and their mutants were measured using a Jasco J-810 spectropolarimeter (Jasco Europe, MI, Italy) in phosphate buffer (pH 7.5) at room temperature using a 0.1-cm path length-stoppered cuvette. Six scans were recorded, averaged and then baseline-subtracted.

### Plant virulence assay

*A. tumefaciens* strains were grown in MG/L liquid medium overnight at 28°C supplemented with appropriate antibiotics. The bacterial cells were collected by centrifugation and re-suspended in a buffer solution consisting of 10 mM MgCl_2_ and 10 mM MES, pH 5.5. Cell concentrations were adjusted to OD_600_ = 0.1. The leaves of *Kalanchoe* plants were wounded with a hypodermic needle and 5 µl of bacterial cell suspension was inoculated onto each wound area. The tumors were photographed at different time points after inoculation.

## Supporting Information

Figure S1 **Electron density map and topology diagram of HEPN domain.** (A) A sample *2Fo-Fc* electron density map (contoured at 1 σ) of HEPN domain of VBP. (B) Topology diagram of the HEPN domain of VBP.
(TIF)

Figure S2 **Comparison of the gel filtration elution profiles of NT domain and HEPN domain of VBP.** NT domain elutes as a single peak (in green) at an elution volume corresponding to an apparent molecular mass of 17 kDa while HEPN domain elutes as a single peak (in brown) at an elution volume corresponding to a molecular mass of 37 kDa. The molecular mass standard is shown in red.
(TIF)

Figure S3 **Substitution of key residues disrupts dimerization in HEPN domain.** (A) Analytical ultra-centrifugation profile of Asp173Asn substituted HEPN domain of VBP. (B) Analytical ultra-centrifugation profile of Lys184Asp substituted HEPN domain of VBP.
(TIF)

Figure S4 **Comparison of the gel filtration elution profiles of HEPN and substituted HEPN domains of VBP.** HEPN domain elutes as a single peak (in brown) at an elution volume corresponding to an apparent molecular mass of 37 kDa while HEPN domain with substitution Asp173Asn (in black)/Lys184Asp (in green)/Asn186Asp (in blue) elutes as a single peak at an elution volume corresponding to a molecular mass of 18.5 kDa. The molecular weight standard is shown in red.
(TIF)

Figure S5 **Substitution of key residues disrupts dimerization in VBP.** (A) Analytical ultra-centrifugation profile of VBP Asp173Asn. (B) Analytical ultra-centrifugation profile of VBP Lys184Asp.
(TIF)

Figure S6 **Comparison of the gel filtration elution profiles VBP and substituted VBP.** VBP domain elutes as a single peak (in green) at an elution volume corresponding to an apparent molecular mass of 75 kDa while VBP Asp173Asn (in brown)/Lys184Asp (in teal)/Asn186Asp (in blue) elutes as a single peak at an elution volume corresponding to a molecular mass of 37.5 kDa. The peak (green) at 670 kDa corresponds to highly aggregated VBP that elutes in the void. The molecular weight standard is shown in red.
(TIF)

Figure S7 **CD spectroscopy of HEPN domain and VBP.** (A) HEPN domain and HEPN domain with substitution Asp173Asn/Lys184Asp/Asn186Asp have identical CD spectra. The graph is color coded. (B) VBP and VBP with substitution Asp173Asn/Lys184Asp/Asn186Asp have identical CD spectra. The graph is color coded.
(TIF)

Figure S8 **VBP is a functional dimer.** The effect of VBP mutations on tumorigenesis. *A. tumefaciens* strains were grown in MG/L medium at 28°C overnight. The cell density was adjusted to 10^8^ cells/ml. The wounds on the *Kalanchoe* leaf were inoculated with this cell suspension (5 µl) of *A.tumefaciens* WT strain or GMV123 strain complemented with plasmid expressing VBP WT, VBP N186D, NT domain or HEPN domain. Only wounds inoculated with *A.tumefaciens* WT strain or GMV123 strain complemented with plasmid expressing VBP WT showed tumor, other wounds showed no tumor clearly indicating that only *Agrobacterium* harboring full length VBP can induce tumor. The tumors shown here were photographed at 35 days to show the growth of tumors over period of time. The particular mutants are labeled at the respective scars.
(TIF)

Figure S9 **Interaction of substituted VBP with AMPPNP (ATP analog) by isothermal titration calorimetry (ITC).** Representative ITC profiles are shown. The upper part of each panel shows the thermogram (thermal power *vs.* time) after baseline correction and the bottom part of each panel shows the binding isotherm (normalized heat *vs.* molar ratio of reactants). (A) Calorimetric titration for VBP K184D. (B) Calorimetric titration for VBP D173N.
(TIF)

Table S1 **Structural homologs of HEPN domain as predicted by DALI search.**
(DOCX)

Table S2 **Thermodynamic parameters for VBP interaction with AMPPNP obtained by ITC.**
(DOCX)

Table S3 **Strains and plasmids used in this study.**
(DOCX)
