# Supplementary figures and images for: Dimerization of VirD2 Binding Protein Is Essential for Agrobacterium Induced Tumor Formation in Plants
Source: PLoS Pathog. 2014 Mar 13;10(3):e1003948. doi: 10.1371/journal.ppat.1003948 (PMC3953389; doi:10.1371/journal.ppat.1003948)

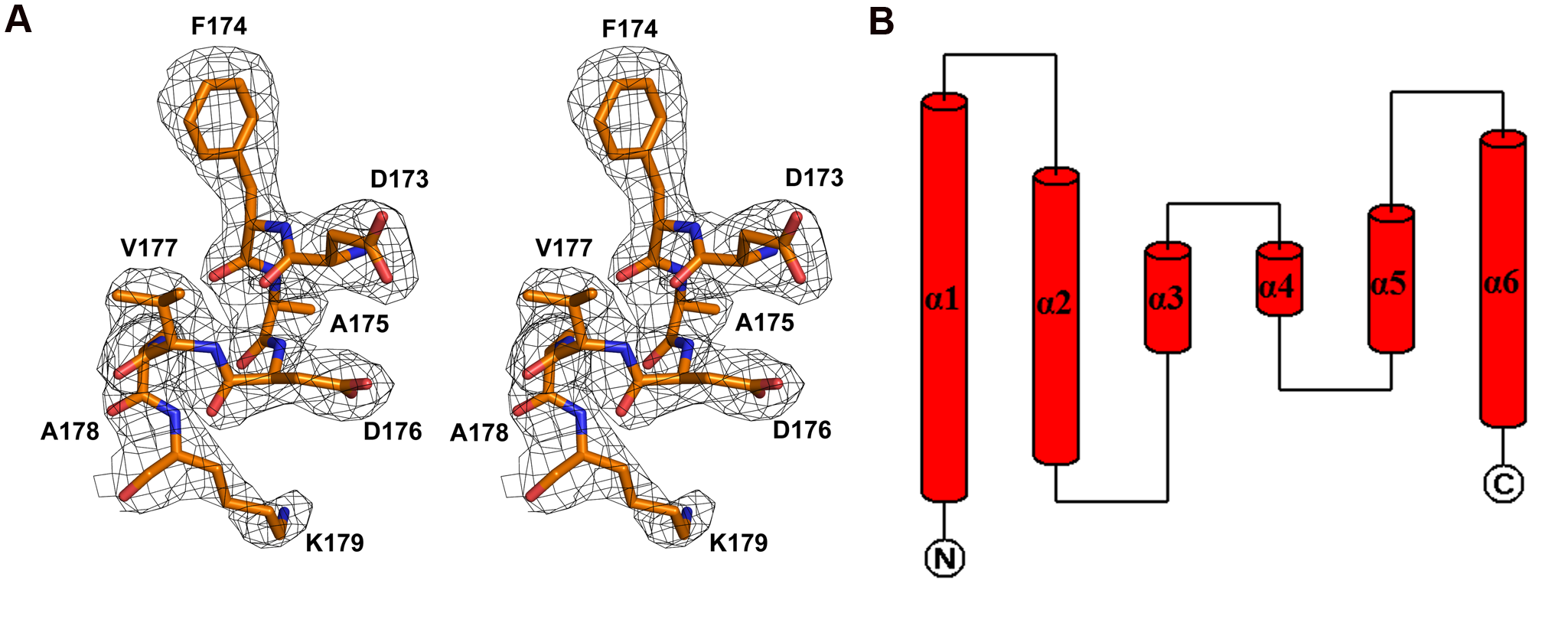

Supplement: Figure S1 — Electron density map and topology diagram of HEPN domain. (A) A sample 2Fo-Fc electron density map (contoured at 1 σ) of HEPN domain of VBP. (B) Topology diagram of the HEPN domain of VBP. (TIF) [file ppat.1003948.s001.tif]

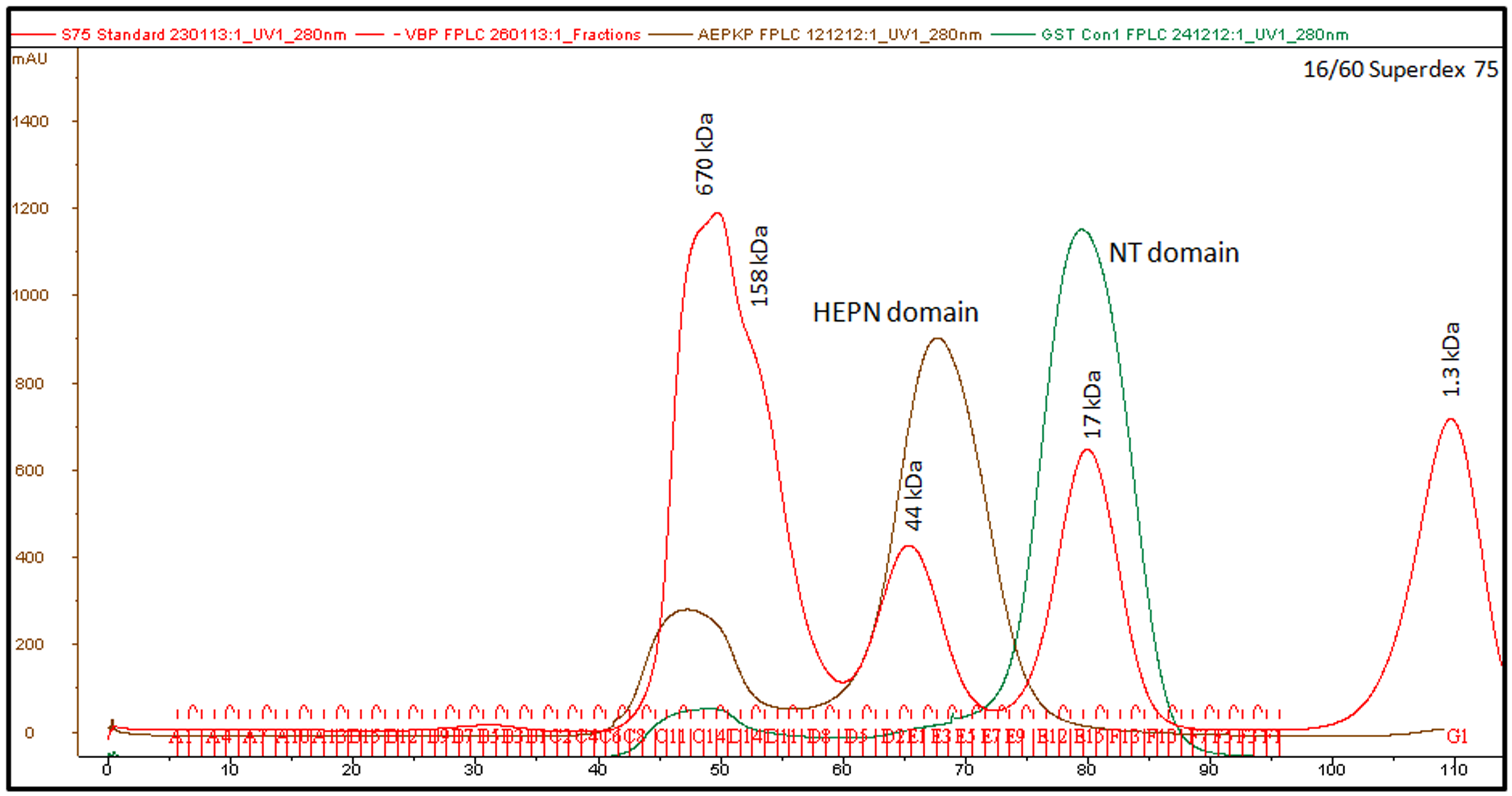

Supplement: Figure S2 — Comparison of the gel filtration elution profiles of NT domain and HEPN domain of VBP. NT domain elutes as a single peak (in green) at an elution volume corresponding to an apparent molecular mass of 17 kDa while HEPN domain elutes as a single peak (in brown) at an elution volume corresponding to a molecular mass of 37 kDa. The molecular mass standard is shown in red. (TIF) [file ppat.1003948.s002.tif]

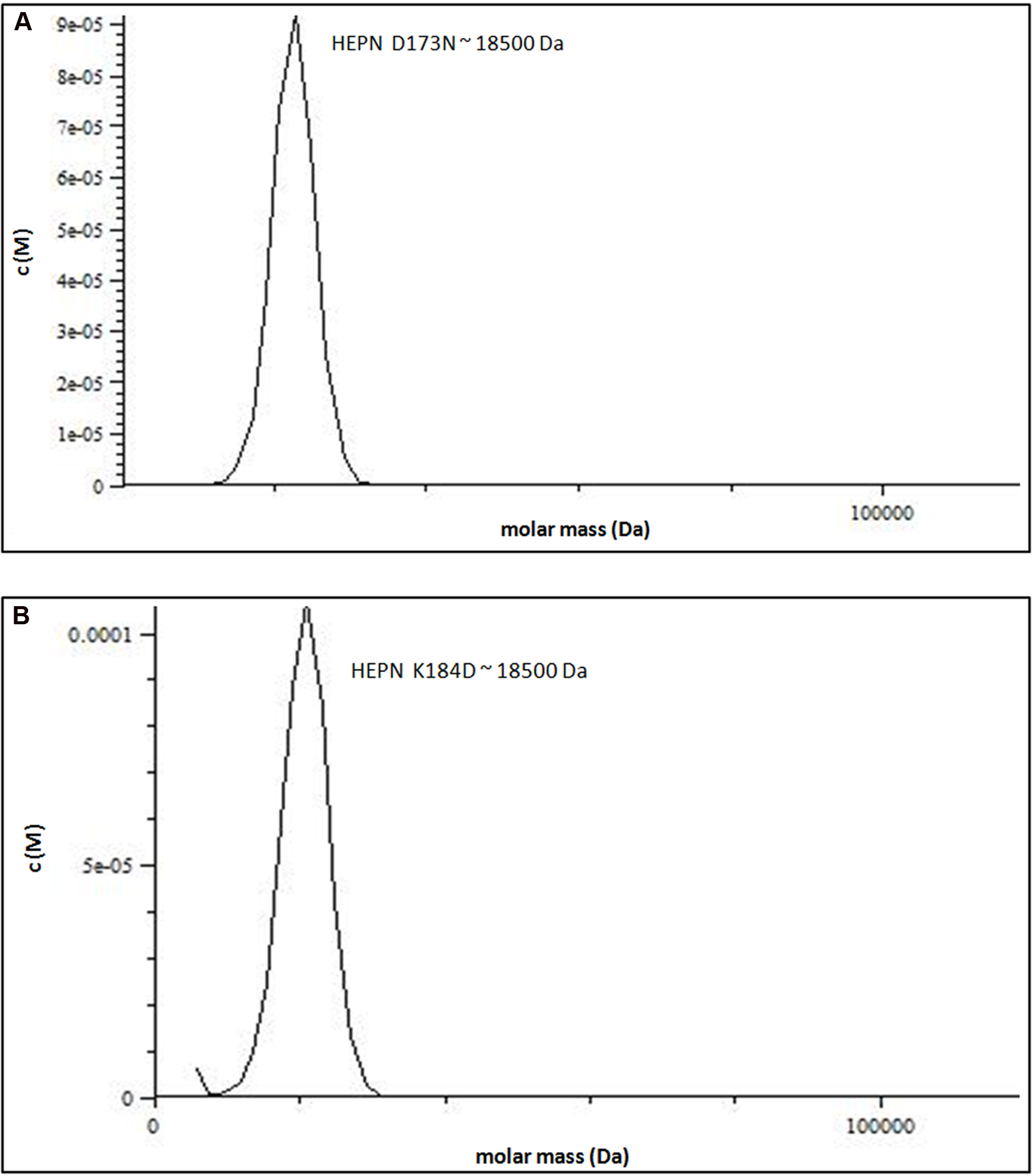

Supplement: Figure S3 — Substitution of key residues disrupts dimerization in HEPN domain. (A) Analytical ultra-centrifugation profile of Asp173Asn substituted HEPN domain of VBP. (B) Analytical ultra-centrifugation profile of Lys184Asp substituted HEPN domain of VBP. (TIF) [file ppat.1003948.s003.tif]

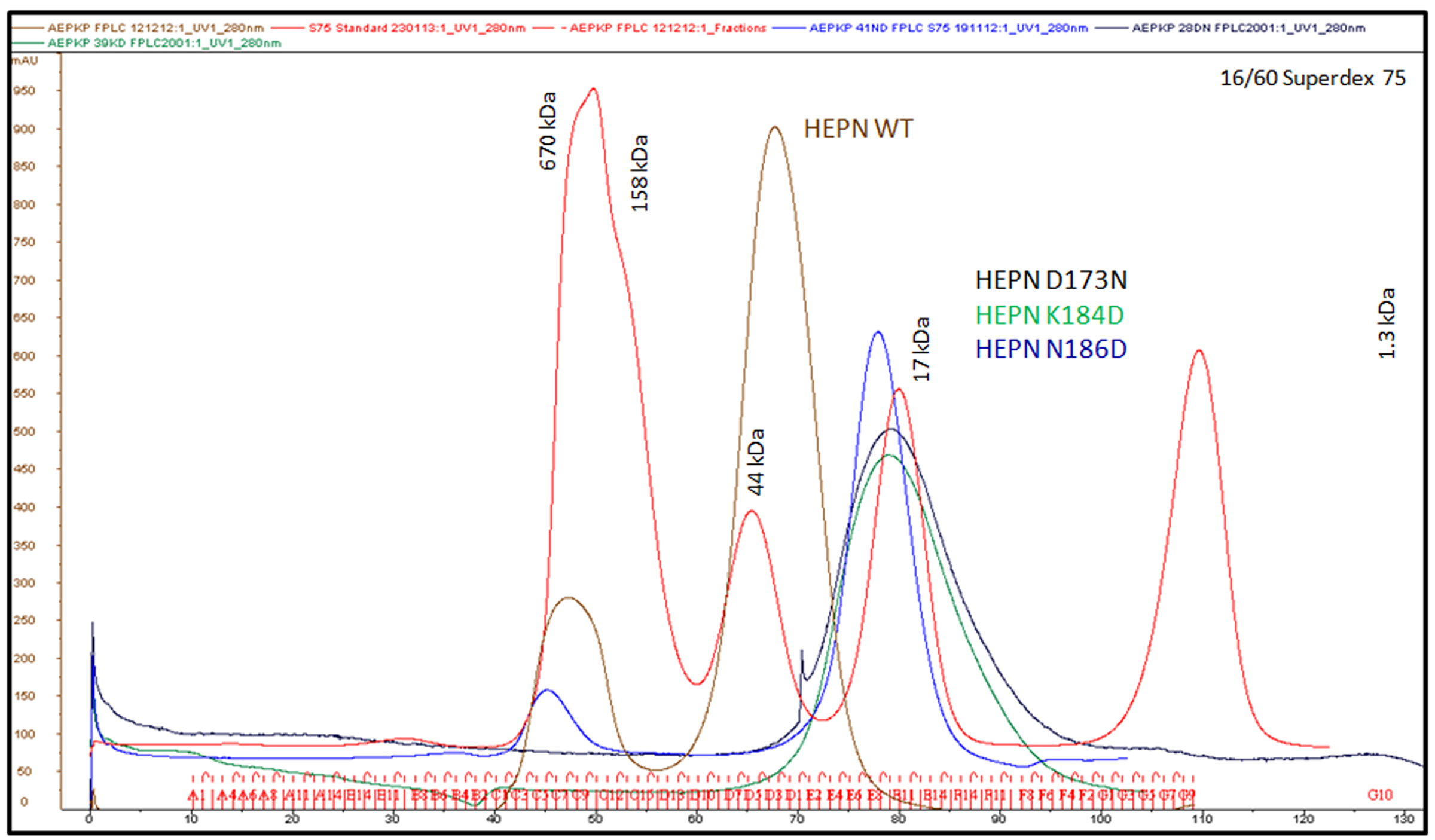

Supplement: Figure S4 — Comparison of the gel filtration elution profiles of HEPN and substituted HEPN domains of VBP. HEPN domain elutes as a single peak (in brown) at an elution volume corresponding to an apparent molecular mass of 37 kDa while HEPN domain with substitution Asp173Asn (in black)/Lys184Asp (in green)/Asn186Asp (in blue) elutes as a single peak at an elution volume corresponding to a molecular mass of 18.5 kDa. The molecular weight standard is shown in red. (TIF) [file ppat.1003948.s004.tif]

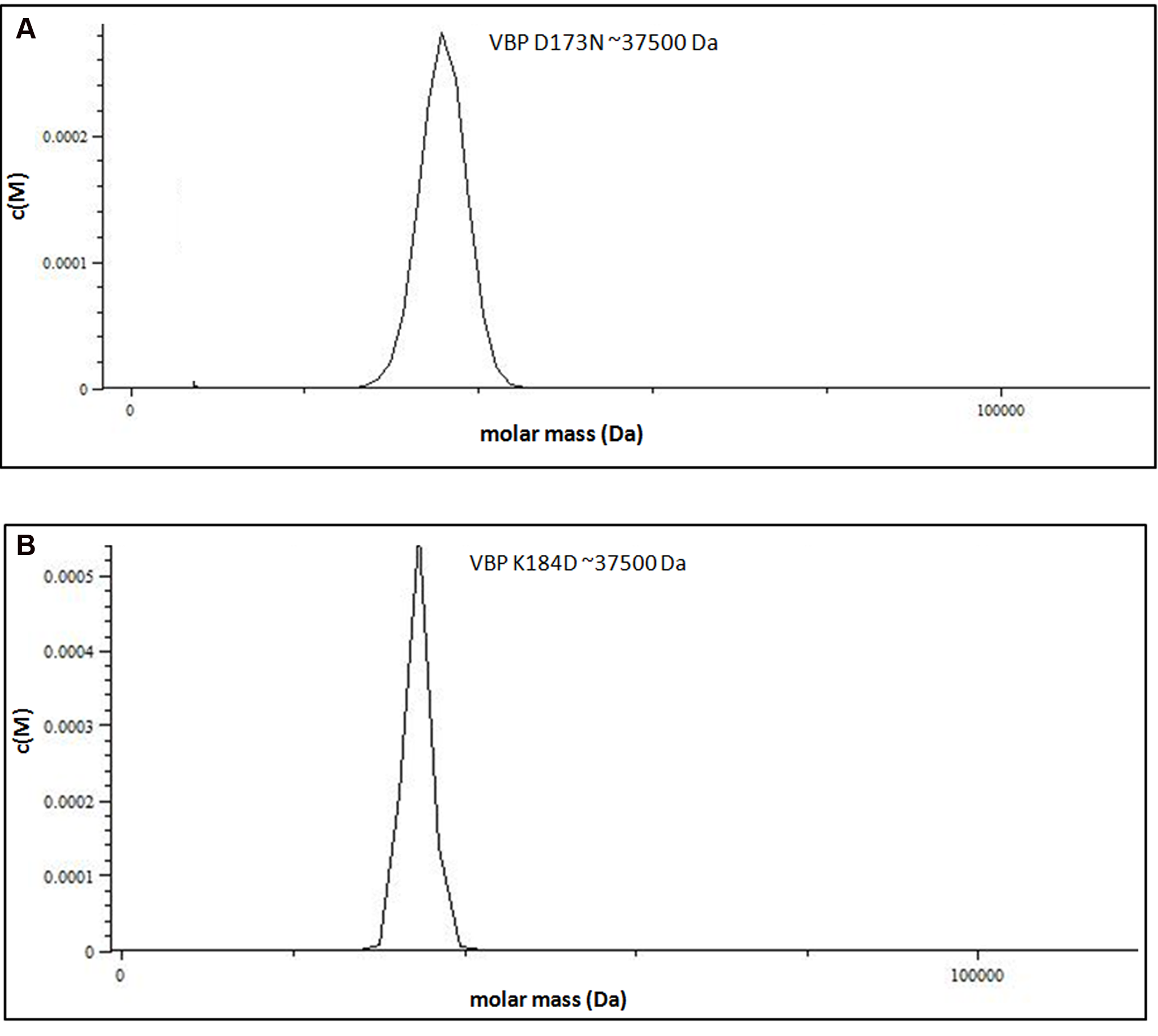

Supplement: Figure S5 — Substitution of key residues disrupts dimerization in VBP. (A) Analytical ultra-centrifugation profile of VBP Asp173Asn. (B) Analytical ultra-centrifugation profile of VBP Lys184Asp. (TIF) [file ppat.1003948.s005.tif]

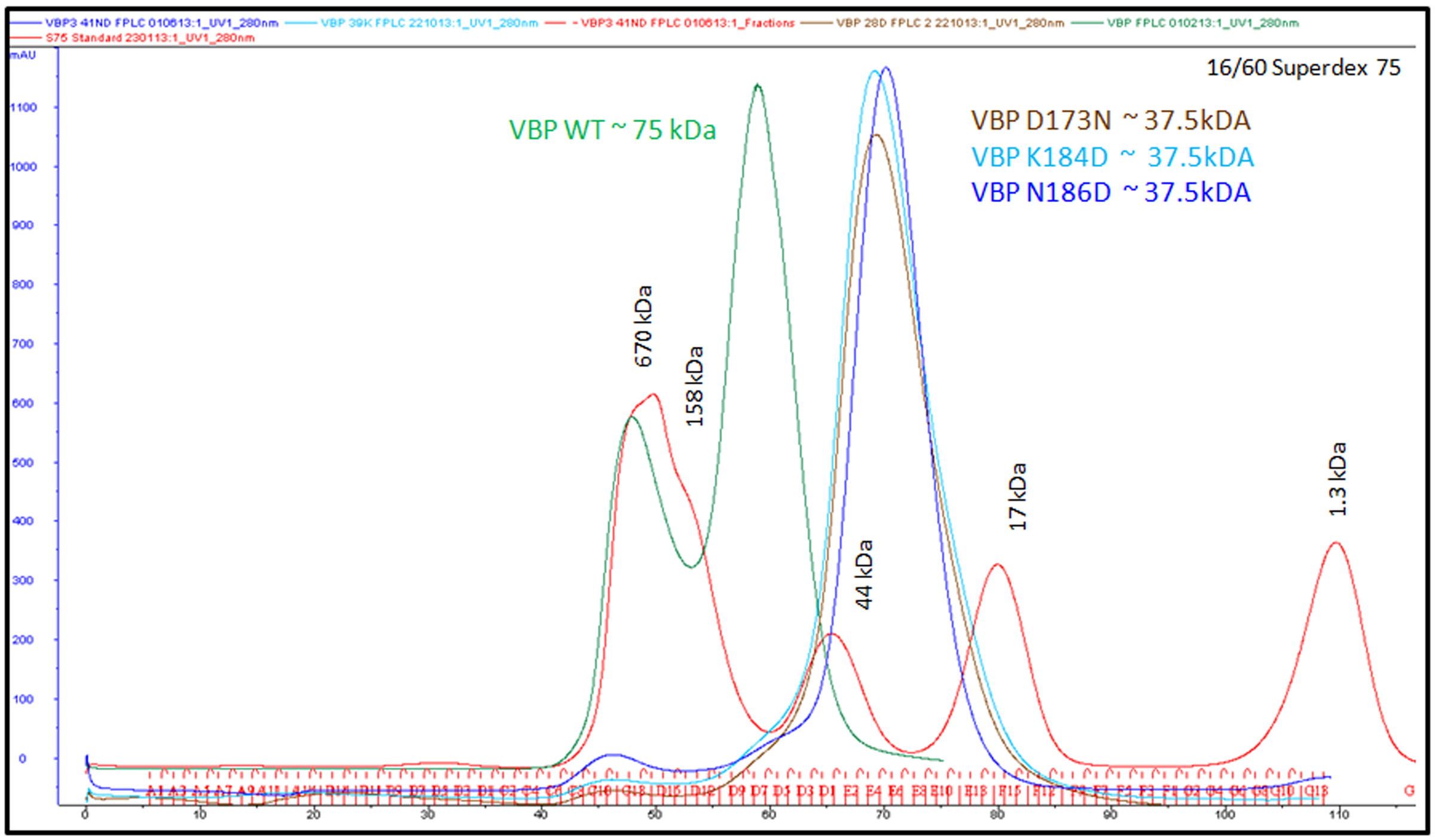

Supplement: Figure S6 — Comparison of the gel filtration elution profiles VBP and substituted VBP. VBP domain elutes as a single peak (in green) at an elution volume corresponding to an apparent molecular mass of 75 kDa while VBP Asp173Asn (in brown)/Lys184Asp (in teal)/Asn186Asp (in blue) elutes as a single peak at an elution volume corresponding to a molecular mass of 37.5 kDa. The peak (green) at 670 kDa corresponds to highly aggregated VBP that elutes in the void. The molecular weight standard is shown in red. (TIF) [file ppat.1003948.s006.tif]

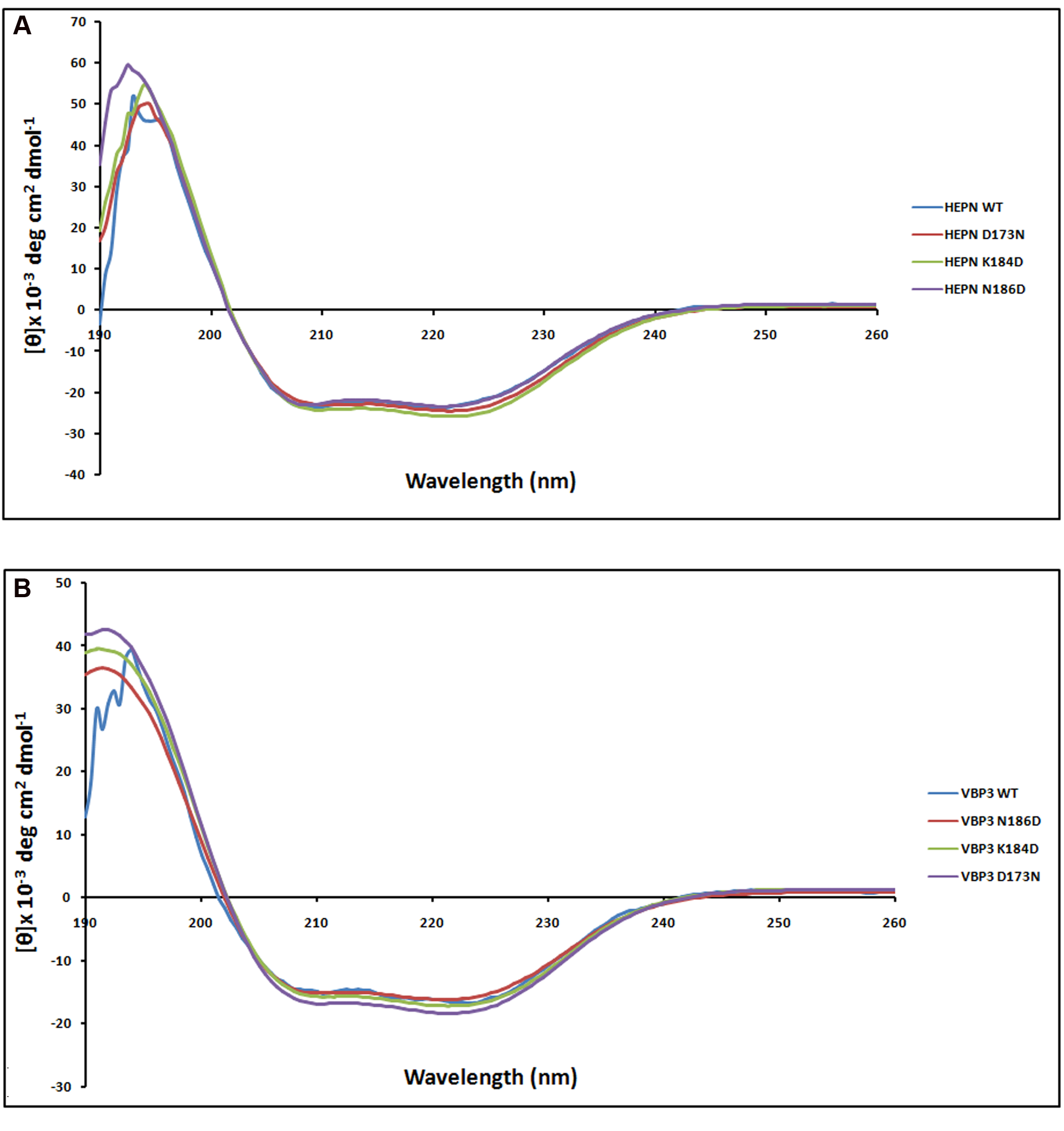

Supplement: Figure S7 — CD spectroscopy of HEPN domain and VBP. (A) HEPN domain and HEPN domain with substitution Asp173Asn/Lys184Asp/Asn186Asp have identical CD spectra. The graph is color coded. (B) VBP and VBP with substitution Asp173Asn/Lys184Asp/Asn186Asp have identical CD spectra. The graph is color coded. (TIF) [file ppat.1003948.s007.tif]

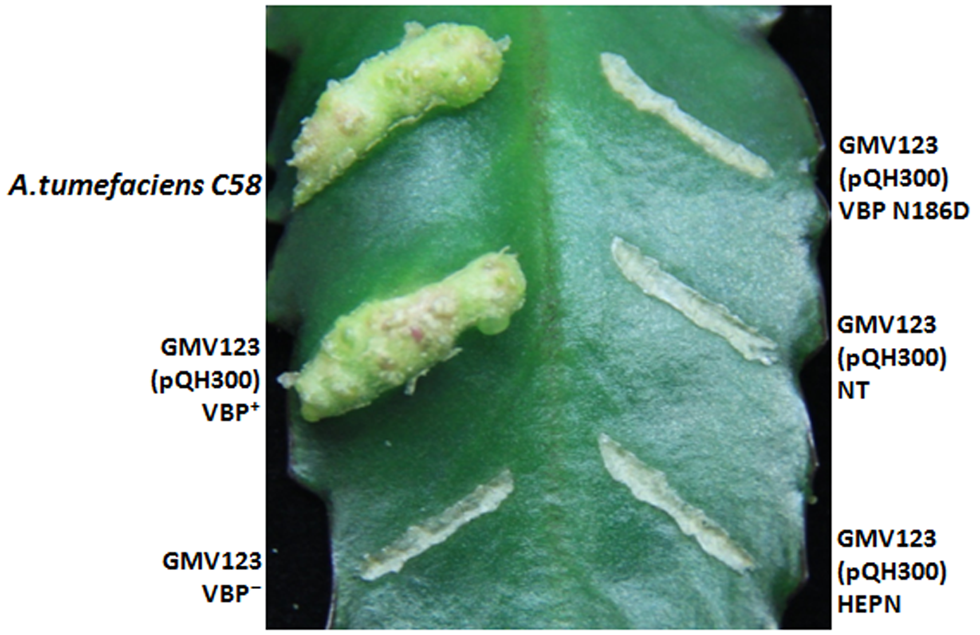

Supplement: Figure S8 — VBP is a functional dimer. The effect of VBP mutations on tumorigenesis. A. tumefaciens strains were grown in MG/L medium at 28°C overnight. The cell density was adjusted to 108 cells/ml. The wounds on the Kalanchoe leaf were inoculated with this cell suspension (5 µl) of A.tumefaciens WT strain or GMV123 strain complemented with plasmid expressing VBP WT, VBP N186D, NT domain or HEPN domain. Only wounds inoculated with A.tumefaciens WT strain or GMV123 strain complemented with plasmid expressing VBP WT showed tumor, other wounds showed no tumor clearly indicating that only Agrobacterium harboring full length VBP can induce tumor. The tumors shown here were photographed at 35 days to show the growth of tumors over period of time. The particular mutants are labeled at the respective scars. (TIF) [file ppat.1003948.s008.tif]

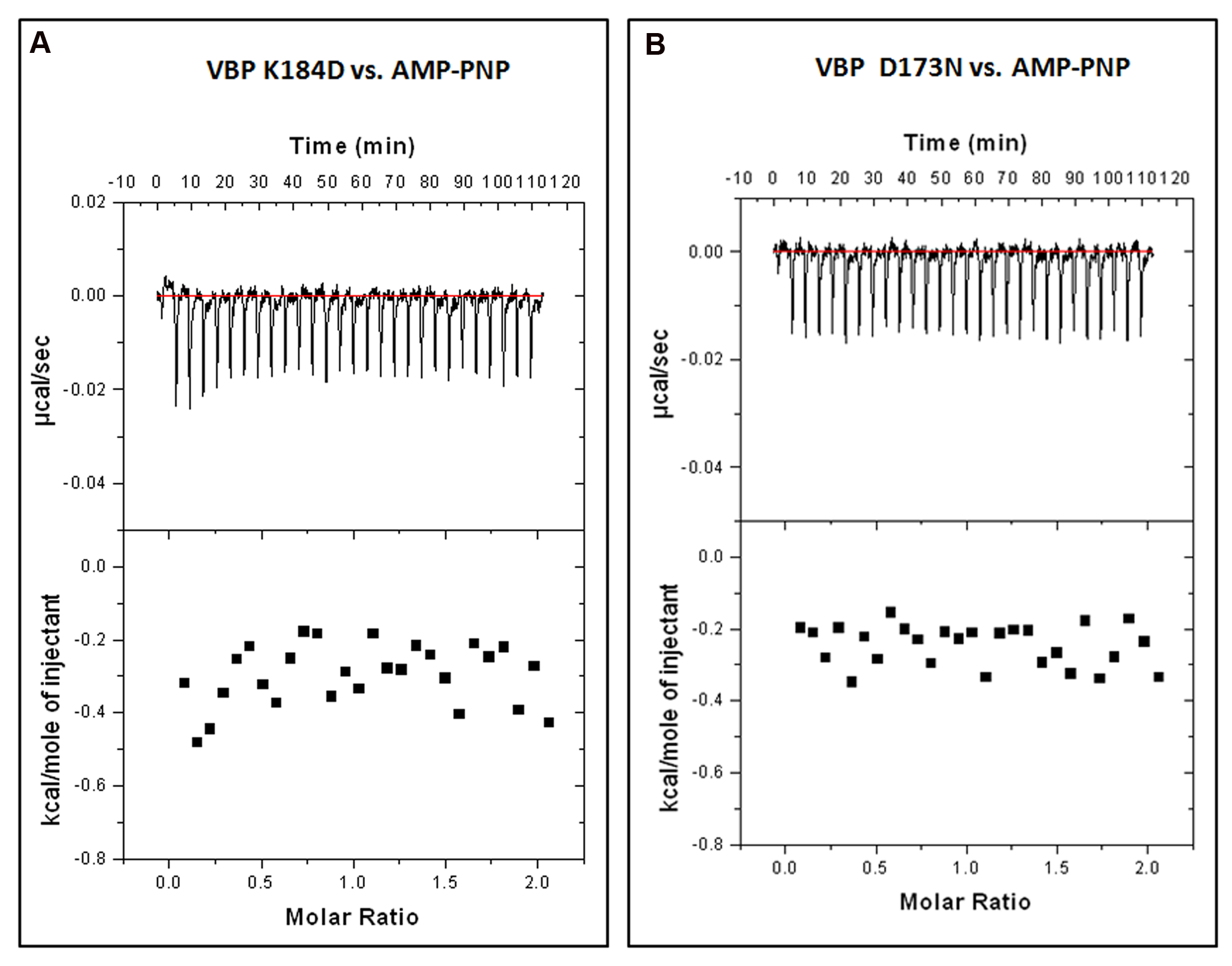

Supplement: Figure S9 — Interaction of substituted VBP with AMPPNP (ATP analog) by isothermal titration calorimetry (ITC). Representative ITC profiles are shown. The upper part of each panel shows the thermogram (thermal power vs. time) after baseline correction and the bottom part of each panel shows the binding isotherm (normalized heat vs. molar ratio of reactants). (A) Calorimetric titration for VBP K184D. (B) Calorimetric titration for VBP D173N. (TIF) [file ppat.1003948.s009.tif]
