# Supplementary material for: Dimerization of VirD2 Binding Protein Is Essential for Agrobacterium Induced Tumor Formation in Plants
Source: PLoS Pathog. 2014 Mar 13;10(3):e1003948. doi: 10.1371/journal.ppat.1003948 (PMC3953389; doi:10.1371/journal.ppat.1003948)
Supplement: Table S1 — Structural homologs of HEPN domain as predicted by DALI search. (DOCX) [file ppat.1003948.s010.docx]

**Table S1.**Structural homologs of HEPN domain as predicted by DALI search.

| **No** | **Pdb** | **z** | **rmsd** | **aa** | **Total residues** | **%id** | **Protein name/detail** |
| --- | --- | --- | --- | --- | --- | --- | --- |
| 1: | 3O10 | 15.0 | 2.0 | 121 | 136 | 16 | SACSIN |
| 2: | 1UFB | 13.5 | 2.2 | 116 | 127 | 16 | TT1696 PROTEIN |
| 3: | 2Q00 | 10.5 | 2.7 | 106 | 122 | 14 | ORF C02003 PROTEIN |
| 4: | 3JYY | 8.9 | 3.0 | 103 | 266 | 7 | LINCOSAMIDE NUCLEOTIDYLTRANSFERASE |
| 5: | 1KNY | 7.6 | 3.3 | 108 | 253 | 7 | KANAMYCIN NUCLEOTIDYLTRANSFERASE |
| 6: | 3AGT | 7.0 | 3.0 | 100 | 133 | 5 | HEMERYTHRIN-LIKE DOMAIN PROTEIN |
