# Supplementary material for: Dimerization of VirD2 Binding Protein Is Essential for Agrobacterium Induced Tumor Formation in Plants
Source: PLoS Pathog. 2014 Mar 13;10(3):e1003948. doi: 10.1371/journal.ppat.1003948 (PMC3953389; doi:10.1371/journal.ppat.1003948)
Supplement: Table S2 — Thermodynamic parameters for VBP interaction with AMPPNP obtained by ITC. (DOCX) [file ppat.1003948.s011.docx]

**Table S2.**Thermodynamic parameters for VBP interaction with AMPPNP obtained by ITC.

|  | **n** | **K_a_ (M^-1^)** | **K_d_ (μM)** | **ΔG (kcal/mol)** | **ΔH (kcal/mol)** | **-TΔS (kcal/mol)** |
| --- | --- | --- | --- | --- | --- | --- |
| **VBP vs. AMPPNP** | **1.10** | **5.0 X 10^5^** | **2.0** | **-7.8** | **-0.5** | **-7.3** |
| **VBP D173N vs. AMPPNP** | **No binding** | | | | | |
| **VBP K184D vs. AMPPNP** | **No binding** | | | | | |
| **VBP N186D vs. AMPPNP** | **No binding** | | | | | |
| **HEPN vs. AMPPNP** | **No binding** | | | | | |

n stoichiometry

K_a_, association constant

K_d_, dissociation constant

ΔG, binding Gibbs energy

ΔH, binding enthalpy

-TΔS, binding entropic contribution
