# Supplementary material for: Dimerization of VirD2 Binding Protein Is Essential for Agrobacterium Induced Tumor Formation in Plants
Source: PLoS Pathog. 2014 Mar 13;10(3):e1003948. doi: 10.1371/journal.ppat.1003948 (PMC3953389; doi:10.1371/journal.ppat.1003948)
Supplement: Table S3 — Strains and plasmids used in this study. (DOCX) [file ppat.1003948.s012.docx]

**Table S3.** Strains and Plasmids used in this study.

| **Strains or plasmids** | **Description*** |
| --- | --- |
| *Agrobacterium tumefaciens C58* | Wild type *Agrobacterium* strain |
| *GMI9017* | C58 cured of pAtC58; lacking *vbp1*; Sm^R^, Sp^R^, Rf^R^ |
| *GMV123* | Derivative of GMI9017 in which *vbp2* and *vbp3* were mutated; Cb^R^ |
| ***E. coli*** |  |
| BL21(DE3) | (IPTG)* induction of T7 polymerase from lacUV5 promoter |
| **Plasmids** |  |
| pET-32a-vbp | pET-32a derivative expressing VBP with N-terminal His_6_ tag |
| pET-32a-vbpD173N | pET-32a derivative expressing VBP D173N with N-terminal His_6_ tag |
| pET-32a-vbpK184D | pET-32a derivative expressing VBP K184D with N-terminal His_6_ tag |
| pET-32a-vbpN186D | pET-32a derivative expressing VBP N186D with N-terminal His_6_ tag |
| pMBP-virD2 | pMAL-c2X derivative expressing MBP-VirD2 under the control of IPTG |
| pGEX-6P1-NTD | pGEX-6P1 derivative expressing GST-NTD(aa 1-145) under the control of IPTG |
| pGEX-6P1-HEPN | pGEX-6P1 derivative expressing GST-HEPN(aa 146-308) under the control of IPTG |
| pGEX-6P1-HEPN D173N | pGEX-6P1 derivative expressing GST-HEPN(aa 146-308) D173N under the control of IPTG |
| pGEX-6P1-HEPN K184D | pGEX-6P1 derivative expressing GST-HEPN(aa 146-308) K184D under the control of IPTG |
| pGEX-6P1-HEPN N186D | pGEX-6P1 derivative expressing GST-HEPN(aa 146-308) N186D under the control of IPTG |
| pQH-300 | Plasmids for expressing proteins in *Agrobacterium* |
| pQH-300-vbp | Plasmids for expressing WT VBP |
| pQH-300-vbpD173N | Plasmids for expressing VBP D173N |
| pQH-300-vbpK184D | Plasmids for expressing VBP K184D |
| pQH-300-vbpN186D | Plasmids for expressing VBP N186D |
| pQH-300-NTD | Plasmids for expressing NT domain |
| pQH-300-HEPN | Plasmids for expressing HEPN domain |

***** *All the strains and plasmids are prepared for this study except for Agrobacterium tumefaciens C58 which is a laboratory collection and E.coli BL21 which is from Invitrogen.*
